# Supplementary figures and images for: Wide range of metabolic adaptations to the acquisition of the Calvin cycle revealed by comparison of microbial genomes
Source: PLoS Comput Biol. 2021 Feb 8;17(2):e1008742. doi: 10.1371/journal.pcbi.1008742 (PMC7895386; doi:10.1371/journal.pcbi.1008742)

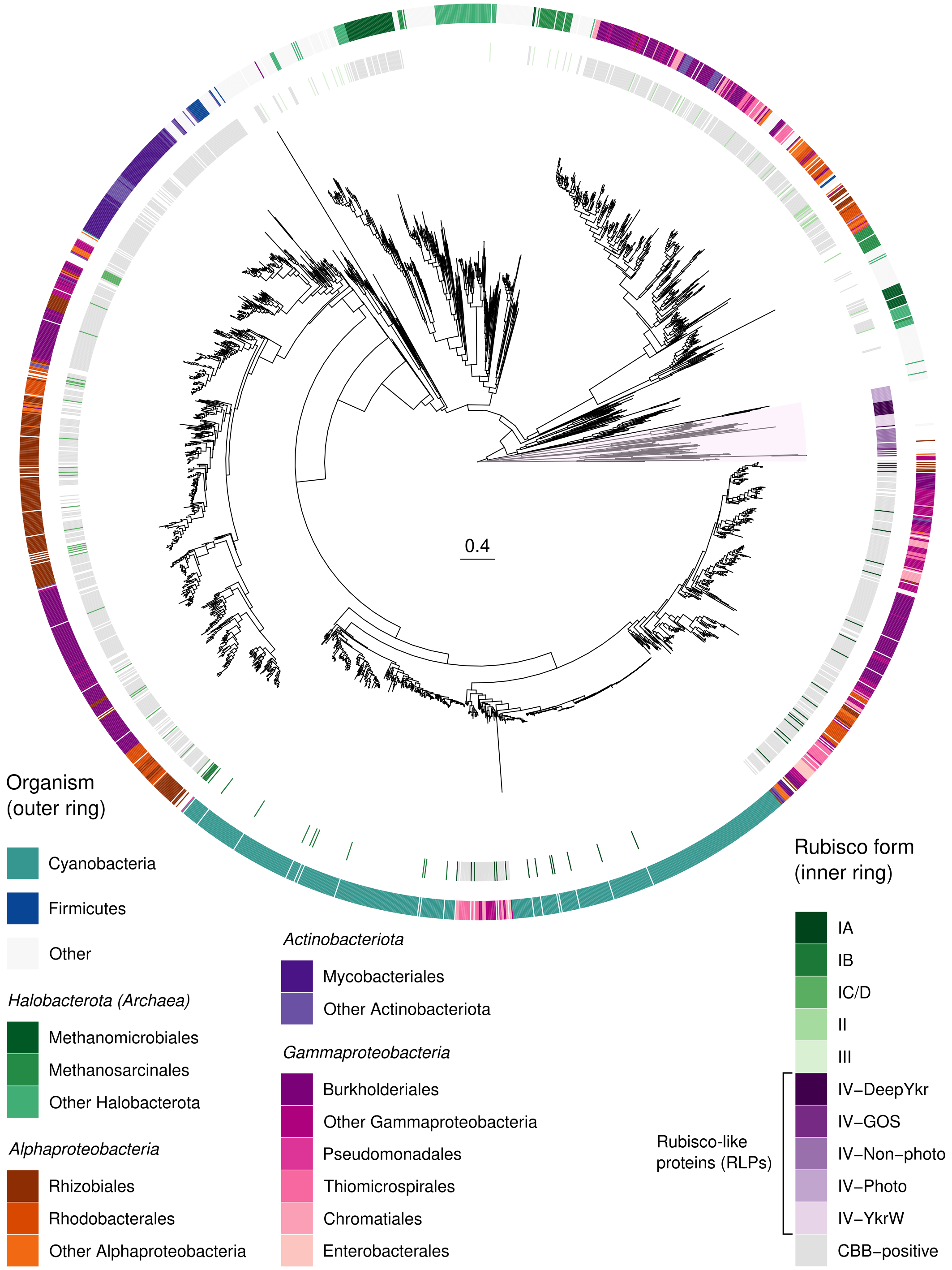

Supplement: S1 Fig — The phylogenetic tree is based on Rubisco sequences identified in ORFs from Genome Taxonomy Database (GTDB) and 181 example Rubisco and Rubisco-like proteins (RLPs) identified by Tabita et al. [19]. Outer ring colors indicate the organism in GTDB carrying each Rubisco ORF, and inner ring colors indicate the Rubisco form. Rubisco sequences from genomes in GTDB identified as CBB-positive, i.e. containing Prk in addition to Rubisco, are indicated in gray in the inner ring. Note that Cyanobacteria, although carrying the Calvin cycle, were not included in the CBB-positive dataset in our analysis. The tree was rooted at the most recent common ancestor of all RLPs, turning the RLPs into an outgroup indicated by light purple shading. The scale bar shows substitutions per site. (PNG) [file pcbi.1008742.s001.png]
