# Supplementary material for: Wide range of metabolic adaptations to the acquisition of the Calvin cycle revealed by comparison of microbial genomes
Source: PLoS Comput Biol. 2021 Feb 8;17(2):e1008742. doi: 10.1371/journal.pcbi.1008742 (PMC7895386; doi:10.1371/journal.pcbi.1008742)
Supplement: S1 Table — The columns contain GTDB accession ID (‘Accession’), species name from GTDB (‘Species’; family name is given within parentheses if used for conclusion), a comment regarding Calvin cycle utilization and autotrophy or other acquired information (‘Comment’), statement about Calvin cycle confirmation (‘CBB’; 1 if likely confirmed, 0 if not likely confirmed, NA if there was no information), and references for the comment (‘Ref.’). There was no information available for seven genomes. For the remaining 43 genomes, 30 (70%) appeared to be likely Calvin-cycle positive genomes, but only one (2%) genome appeared unlikely to be Calvin-cycle positive given its status as a human pathogen (Mycolicibacterium mageritense). (PDF) [file pcbi.1008742.s002.pdf]

S1 Table. Random sample of 50 CBB-positive genomes with literature references supporting or opposing Calvin cycle utilization and autotrophy.

Accession	Species	Comment	CBB	Ref.
GB_GCA_003137095.1	Bog-1198 sp003137095	No information	NA	NA
RS_GCF_000698555.1	*Caballeronia grimmiae*	Isolated from moss	0	[1,2]
GB_GCA_003244105.1	*Eremiobacter* sp003244105	Calvin cycle observed but not confirmed operational	1	[3]
RS_GCF_003057965.1	*Thermodesulfobium acidiphilum*	Chemolithoautotrophic	1	[4]
GB_GCA_002726415.1	GCA-2726415 sp002726415 (Acidiferrobacteraceae)	Chemolithoautotrophic	1	[5]
RS_GCF_000012425.1	*Dechloromonas aromatica*	Calvin cycle observed but not confirmed operational	1	[6]
RS_GCF_000612825.1	*Mycolicibacterium mageritense*	Human pathogen; predominantly environmental	0	[7,8]
RS_GCF_000502875.1	*Mesorhizobium* sp000502875	No information	NA	NA
GB_GCA_002366795.1	UBA3069 sp002366795 (Rhodobacteraceae)	Phototrophic; CBB-positive or -negative	1	[9,10]
GB_GCA_003165655.1	Palsa-465 sp003165655	No information	NA	NA
GB_GCA_900105125.1	*Bradyrhizobium canariense*_A	Nodulates genistoid legumes of Canary Islands	0	[11]
RS_GCF_000227215.1	*Acidithiobacillus thiooxidans*	Sulfur oxidizer that uses Calvin cycle	1	[12]
RS_GCF_001612825.1	*Nocardia caishijiensis*	Endophyte	0	[13]
RS_GCF_002903155.1	*Sulfobacillus* sp002903155	Sulfur oxidizer that uses Calvin cycle	1	[14]
RS_GCF_000297055.2	*Sulfuricella denitrificans*	Sulfur oxidizer that uses Calvin cycle	1	[15]
RS_GCF_001558155.1	*Pseudoruegeria*_C *sabulilitoris*	Isolated from seashore sand; Phototrophic; CBB-positive or -negative	1	[9,10,16]
RS_GCF_000196675.1	*Pseudonocardia dioxanivorans*	Chemolithoautotrophic; Uses Calvin cycle	1	[17,18]
RS_GCF_000701585.1	*Acidiphilium angustum*	Quasiphotosynthetic/Photoheterotrophic	0	[19,20]
RS_GCF_000227665.2	*Thiomicrospira aerophila*	Sulfur-oxidizing chemolithoautotroph with Rubisco	1	[21]
GB_GCA_002083395.1	*Nitrosomonas* sp002083395	Ammonia-oxidizing bacterium that uses Calvin cycle	1	[22]
RS_GCF_001613365.1	*Nocardia xishanensis*	Isolated from soil	0	[23]
GB_GCA_003221005.1	AG25 sp003221005	No information	NA	NA
GB_GCA_003138975.1	*Sulfotelmatobacter* sp003138975	Potentially sulfur- and hydrogen-oxidizing	1	[24]
RS_GCF_900104745.1	*Actinopolymorpha singaporensis*	Isolated from tropical rainforest	0	[25]
RS_GCF_001458355.1	*Thalassobius gelatinovorus*	Chemo-organotroph; Phototrophic; CBB-positive or -negative	1	[9,10,26]
UBA9297	*Methanothrix*_A sp9297u	Rubisco-mediated CO2 reduction	1	[27]
RS_GCF_900155475.1	*Methylomagnum ishizawai*	RuMP and RuBP (Calvin cycle) pathways active	1	[28]
RS_GCF_000013085.1	*Rhodospirillum rubrum*	Photoautotroph that uses the Calvin cycle	1	[9]
RS_GCF_900104845.1	*Paraburkholderia*_B *caballeronis*	Tomato-associated	0	[29]

RS_GCF_001583505.1	*Marichromatium gracile*	Sulfur-oxidation and denitrification	1	[30]
RS_GCF_003053725.1	*Rhodovulum kholense*	Photolithoautotrophic; Phototrophic; CBB-positive or -negative	1	[9,10,31]
GB_GCA_002695745.2	GCA-002705045 sp002695745 (Rhodobacteraceae)	Phototrophic; CBB-positive or -negative	1	[9,10]
GB_GCA_001512375.1	*Methanoculleus thermohydrogenotrophicum*	Hydrogenotrophic methanogen	0	[32]
GB_GCA_003104975.1	*Methylomirabilis* sp003104975	Uses Calvin cycle	1	[33]
GB_GCA_001447805.1	*Tenderia electrophaga*	Electroautotroph that uses the Calvin cycle	1	[34,35]
RS_GCF_900129505.1	*Bradyrhizobium erythrophlei*_D	Isolated from ironwood nodules	0	[36]
RS_GCF_000787695.1	*Paracoccus* sp000787695 (Rhodobacteraceae)	Phototrophic; CBB-positive or -negative	1	[9,10,37]
RS_GCF_001295865.1	*Rubrivivax* sp001295865	Chemolithoautotrophic; Uses Calvin cycle	1	[38]
RS_GCF_000304355.2	*Methanoculleus bourgensis*	Hydrogenotrophic methanogen	0	[39]
GB_GCA_003233235.1	SZUA-26 sp003233235 (Mariprofundaceae)	Iron-oxidizing chemolithoautotroph; Has Calvin cycle genes	1	[40]
GB_GCA_003446655.1	*Thauera* sp003446655	Chemolithoautotrophic	1	[41]
GB_GCA_002839575.1	*Methanoculleus* sp002839575	Hydrogenotrophic methanogen	0	[32,39]
GB_GCA_003160215.1	Bog-257 sp003160215	No information	NA	NA
GB_GCA_001899775.1	*Thiobacillus* sp001899775	Chemolithoautotrophic; Uses Calvin cycle	1	[42,43]
GB_GCA_003138855.1	Bog-756 sp003138855	No information	NA	NA
GB_GCA_002256995.1	*Thiobacillus* sp002256995	Chemolithoautotrophic; Uses Calvin cycle	1	[42,43]
GB_GCA_003157035.1	Fen-999 sp003157035 (Rhodocyclaceae)	Some family members are photoautotrophic	1	[21]
GB_GCA_003228405.1	SZUA-229 sp003228405	No information	NA	NA
RS_GCF_000243255.1	*Methanoplanus limicola*	Methanogen	0	[44]
GB_GCA_002256785.1	*Thiobacillus* sp002256785	Chemolithoautotrophic; Uses Calvin cycle	1	[42,43]

The columns contain GTDB accession ID ('Accession'), species name from GTDB ('Species'; family name is given within parentheses if used for conclusion), a comment regarding Calvin cycle utilization and autotrophy or other acquired information ('Comment'), statement about Calvin cycle confirmation ('CBB'; 1 if likely confirmed, 0 if not likely confirmed, NA if there was no information), and references for the comment ('Ref.'). There was no information available for seven genomes. For the remaining 43 genomes, 30 (70%) appeared to be likely Calvin-cycle positive genomes, but only one (2%) genome appeared unlikely to be Calvin-cycle positive given its status as a human pathogen (*Mycolicibacterium mageritense*).

References

1. Peeters C, Meier-Kolthoff JP, Verheyde B, De Brandt E, Cooper VS, Vandamme P. Phylogenomic Study of Burkholderia glathei-like Organisms, Proposal of 13 Novel Burkholderia Species and Emended Descriptions of Burkholderia sordidicola, Burkholderia zhejiangensis, and Burkholderia grimmiae. Front Microbiol. 2016;7. doi:10.3389/fmicb.2016.00877

2. Tian Y, Kong BH, Liu SL, Li CL, Yu R, Liu L, et al. Burkholderia grimmiae sp. nov., isolated from a xerophilous moss (Grimmia montana). Int J Syst Evol Microbiol. 2013;63: 2108–2113. doi:10.1099/ijs.0.045492-0

3. Ward LM, Cardona T, Holland-Moritz H. Evolutionary Implications of Anoxygenic Phototrophy in the Bacterial Phylum Candidatus Eremiobacterota (WPS-2). Front Microbiol. 2019;10. doi:10.3389/fmicb.2019.01658

4. Frolov EN, Kublanov IV, Toshchakov SV, Samarov NI, Novikov AA, Lebedinsky AV, et al. Thermodesulfobium acidiphilum sp. nov., a thermoacidophilic, sulfate-reducing, chemoautotrophic bacterium from a thermal site. Int J Syst Evol Microbiol. 2017;67: 1482–1485. doi:10.1099/ijsem.0.001745

5. Issotta F, Moya-Beltrán A, Mena C, Covarrubias PC, Thyssen C, Bellenberg S, et al. Insights into the biology of acidophilic members of the Acidiferrobacteraceae family derived from comparative genomic analyses. Res Microbiol. 2018;169: 608–617. doi:10.1016/j.resmic.2018.08.001

6. Salinero KK, Keller K, Feil WS, Feil H, Trong S, Di Bartolo G, et al. Metabolic analysis of the soil microbe Dechloromonas aromatica str. RCB: indications of a surprisingly complex life-style and cryptic anaerobic pathways for aromatic degradation. BMC Genomics. 2009;10: 351. doi:10.1186/1471-2164-10-351

7. Wallace RJ, Brown-Elliott BA, Hall L, Roberts G, Wilson RW, Mann LB, et al. Clinical and Laboratory Features of Mycobacterium mageritense. J Clin Microbiol. 2002;40: 2930–2935. doi:10.1128/JCM.40.8.2930-2935.2002

8. Gupta RS, Lo B, Son J. Phylogenomics and Comparative Genomic Studies Robustly Support Division of the Genus Mycobacterium into an Emended Genus Mycobacterium and Four Novel Genera. Front Microbiol. 2018;9. doi:10.3389/fmicb.2018.00067

9. Gordon GC, McKinlay JB. Calvin Cycle Mutants of Photoheterotrophic Purple Nonsulfur Bacteria Fail To Grow Due to an Electron Imbalance Rather than Toxic Metabolite Accumulation. J Bacteriol. 2014;196: 1231–1237. doi:10.1128/JB.01299-13

10. Brinkmann H, Göker M, Koblížek M, Wagner-Döbler I, Petersen J. Horizontal operon transfer, plasmids, and the evolution of photosynthesis in Rhodobacteraceae. ISME J. 2018;12: 1994–2010. doi:10.1038/s41396-018-0150-9

11. Vinuesa P, León-Barrios M, Silva C, Willems A, Jarabo-Lorenzo A, Pérez-Galdona R, et al. Bradyrhizobium canariense sp. nov., an acid-tolerant endosymbiont that nodulates endemic

genistoid legumes (Papilionoideae: Genisteae) from the Canary Islands, along with Bradyrhizobium japonicum bv. genistearum, Bradyrhizobium genospecies alpha and Bradyrhizobium genospecies beta. Int J Syst Evol Microbiol. 2005;55: 569–575. doi:10.1099/ijs.0.63292-0

12. Yang L, Zhao D, Yang J, Wang W, Chen P, Zhang S, et al. Acidithiobacillus thiooxidans and its potential application. Appl Microbiol Biotechnol. 2019;103: 7819–7833. doi:10.1007/s00253-019-10098-5

13. Tanvir R, Sajid I, Hasnain S, Kulik A, Grond S. Rare actinomycetes Nocardia caishijiensis and Pseudonocardia carboxydivorans as endophytes, their bioactivity and metabolites evaluation. Microbiol Res. 2016;185: 22–35. doi:10.1016/j.micres.2016.01.003

14. Justice NB, Norman A, Brown CT, Singh A, Thomas BC, Banfield JF. Comparison of environmental and isolate Sulfobacillus genomes reveals diverse carbon, sulfur, nitrogen, and hydrogen metabolisms. BMC Genomics. 2014;15: 1107. doi:10.1186/1471-2164-15-1107

15. Kojima H, Fukui M. Sulfuricella denitrificans gen. nov., sp. nov., a sulfur-oxidizing autotroph isolated from a freshwater lake. Int J Syst Evol Microbiol. 2010;60: 2862–2866. doi:10.1099/ijs.0.016980-0

16. Park S, Jung Y-T, Won S-M, Yoon J-H. Pseudoruegeria sabulilitoris sp. nov., isolated from seashore sand. Int J Syst Evol Microbiol. 2014;64: 3276–3281. doi:10.1099/ijs.0.066258-0

17. Mahendra S, Alvarez-Cohen L. Pseudonocardia dioxanivorans sp. nov., a novel actinomycete that grows on 1,4-dioxane. Int J Syst Evol Microbiol. 2005;55: 593–598. doi:10.1099/ijs.0.63085-0

18. Sales CM, Mahendra S, Grostern A, Parales RE, Goodwin LA, Woyke T, et al. Genome Sequence of the 1,4-Dioxane-Degrading Pseudonocardia dioxanivoransStrain CB1190. J Bacteriol. 2011;193: 4549–4550. doi:10.1128/JB.00415-11

19. Kishimoto N, Fukaya F, Inagaki K, Sugio T, Tanaka H, Tano T. Distribution of bacteriochlorophyll a among aerobic and acidophilic bacteria and light-enhanced CO2-incorporation in Acidiphilium rubrum. FEMS Microbiol Ecol. 1995;16: 291–296. doi:10.1111/j.1574-6941.1995.tb00293.x

20. Wichlacz PL, Unz RF, Langworthy TA. Acidiphilium angustum sp. nov., Acidiphilium facilis sp. nov., and Acidiphilium rubrum sp. nov.: Acidophilic Heterotrophic Bacteria Isolated from Acidic Coal Mine Drainage. Int J Syst Evol Microbiol. 1986;36: 197–201. doi:10.1099/00207713-36-2-197

21. Boden R, Scott KM, Williams J, Russel S, Antonen K, Rae AW, et al. An evaluation of Thiomicrospira, Hydrogenovibrio and Thioalkalimicrobium: reclassification of four species of Thiomicrospira to each Thiomicrorhabdus gen. nov. and Hydrogenovibrio, and reclassification of all four species of Thioalkalimicrobium to Thiomicrospira. Int J Syst Evol Microbiol. 2017;67: 1140–1151. doi:10.1099/ijsem.0.001855

22. Chain P, Lamerdin J, Larimer F, Regala W, Lao V, Land M, et al. Complete Genome Sequence of the Ammonia-Oxidizing Bacterium and Obligate Chemolithoautotroph Nitrosomonas europaea. J Bacteriol. 2003;185: 2759–2773. doi:10.1128/JB.185.9.2759-2773.2003

23. Zhang J, Liu Z, Goodfellow M. Nocardia xishanensis sp. nov., a novel actinomycete isolated from soil. Int J Syst Evol Microbiol. 2004;54: 2301–2305. doi:10.1099/ijs.0.63133-0

24. Hausmann B, Pelikan C, Herbold CW, Köstlbacher S, Albertsen M, Eichorst SA, et al. Peatland Acidobacteria with a dissimilatory sulfur metabolism. ISME J. 2018;12: 1729–1742. doi:10.1038/s41396-018-0077-1

25. Wang YM, Zhang ZS, Xu XL, Ruan JS, Wang Y. Actinopolymorpha singaporensis gen. nov., sp. nov., a novel actinomycete from the tropical rainforest of Singapore. Int J Syst Evol Microbiol. 2001;51: 467–473. doi:10.1099/00207713-51-2-467

26. Arahal DR, Macián MC, Garay E, Pujalte MJ. Thalassobius mediterraneus gen. nov., sp. nov., and reclassification of Ruegeria gelatinovorans as Thalassobius gelatinovorus comb. nov. Int J Syst Evol Microbiol. 2005;55: 2371–2376. doi:10.1099/ijs.0.63842-0

27. Yang P, Tan G-YA, Aslam M, Kim J, Lee P-H. Metatranscriptomic evidence for classical and RuBisCO-mediated CO 2 reduction to methane facilitated by direct interspecies electron transfer in a methanogenic system. Sci Rep. 2019;9: 4116. doi:10.1038/s41598-019-40830-0

28. Khalifa A, Lee CG, Ogiso T, Ueno C, Dianou D, Demachi T, et al. Methylomagnum ishizawai gen. nov., sp. nov., a mesophilic type I methanotroph isolated from rice rhizosphere. Int J Syst Evol Microbiol. 2015;65: 3527–3534. doi:10.1099/ijsem.0.000451

29. Rojas-Rojas FU, Tapia-García EY, Maymon M, Humm E, Huntemann M, Clum A, et al. Draft genome of Paraburkholderia caballeronis TNe-841T, a free-living, nitrogen-fixing, tomato plant-associated bacterium. Stand Genomic Sci. 2017;12: 80. doi:10.1186/s40793-017-0294-7

30. Zhu B, Zhang X, Zhao C, Chen S, Yang S. Comparative genome analysis of marine purple sulfur bacterium Marichromatium gracile YL28 reveals the diverse nitrogen cycle mechanisms and habitat-specific traits. Sci Rep. 2018;8: 17803. doi:10.1038/s41598-018-36160-2

31. Kumar PA, Aparna P, Srinivas TNR, Sasikala Ch, Ramana ChV. Rhodovulum kholense sp. nov. Int J Syst Evol Microbiol. 2008;58: 1723–1726. doi:10.1099/ijs.0.65620-0

32. Kougias PG, Campanaro S, Treu L, Zhu X, Angelidaki I. A novel archaeal species belonging to Methanoculleus genus identified via de-novo assembly and metagenomic binning process in biogas reactors. Anaerobe. 2017;46: 23–32. doi:10.1016/j.anaerobe.2017.02.009

33. Versantvoort W, Guerrero-Cruz S, Speth DR, Frank J, Gambelli L, Cremers G, et al. Comparative Genomics of Candidatus Methylomirabilis Species and Description of Ca. Methylomirabilis Lanthanidiphila. Front Microbiol. 2018;9. doi:10.3389/fmicb.2018.01672

34. Eddie BJ, Wang Z, Hervey WJ, Leary DH, Malanoski AP, Tender LM, et al. Metatranscriptomics Supports the Mechanism for Biocathode Electroautotrophy by "Candidatus Tenderia electrophaga." mSystems. 2017;2. doi:10.1128/mSystems.00002-17

35. Eddie BJ, Wang Z, Malanoski AP, Hall RJ, Oh SD, Heiner C, et al. 'Candidatus Tenderia electrophaga', an uncultivated electroautotroph from a biocathode enrichment. Int J Syst Evol Microbiol. 2016;66: 2178–2185. doi:10.1099/ijsem.0.001006

36. Yao Y, Sui XH, Zhang XX, Wang ET, Chen WX. Bradyrhizobium erythrophlei sp. nov. and Bradyrhizobium ferriligni sp. nov., isolated from effective nodules of Erythrophleum fordii. Int J Syst Evol Microbiol. 2015;65: 1831–1837. doi:10.1099/ijs.0.000183

37. Baker SC, Ferguson SJ, Ludwig B, Page MD, Richter O-MH, Spanning RJM van. Molecular Genetics of the GenusParacoccus: Metabolically Versatile Bacteria with Bioenergetic Flexibility. Microbiol Mol Biol Rev. 1998;62: 1046–1078. doi:10.1128/MMBR.62.4.1046-1078.1998

38. Wawrousek K, Noble S, Korlach J, Chen J, Eckert C, Yu J, et al. Genome Annotation Provides Insight into Carbon Monoxide and Hydrogen Metabolism in Rubrivivax gelatinosus. PLOS ONE. 2014;9: e114551. doi:10.1371/journal.pone.0114551

39. Maus I, Wibberg D, Stantscheff R, Eikmeyer F-G, Seffner A, Boelter J, et al. Complete Genome Sequence of the Hydrogenotrophic, Methanogenic Archaeon Methanoculleus bourgensis Strain MS2T, Isolated from a Sewage Sludge Digester. J Bacteriol. 2012;194: 5487–5488. doi:10.1128/JB.01292-12

40. Emerson D, Rentz JA, Lilburn TG, Davis RE, Aldrich H, Chan C, et al. A Novel Lineage of Proteobacteria Involved in Formation of Marine Fe-Oxidizing Microbial Mat Communities. PLOS ONE. 2007;2: e667. doi:10.1371/journal.pone.0000667

41. Jiang K, Sanseverino J, Chauhan A, Lucas S, Copeland A, Lapidus A, et al. Complete genome sequence of Thauera aminoaromatica strain MZ1T. Stand Genomic Sci. 2012;6: 325. doi:10.4056/sigs.2696029

42. Gale NL, Beck JV. Evidence for the Calvin Cycle and Hexose Monophosphate Pathway in Thiobacillus ferrooxidans. J Bacteriol. 1967;94: 1052–1059.

43. Nemati M, Harrison STL, Hansford GS, Webb C. Biological oxidation of ferrous sulphate by Thiobacillus ferrooxidans: a review on the kinetic aspects. Biochem Eng J. 1998;1: 171–190. doi:10.1016/S1369-703X(98)00006-0

44. Göker M, Lu M, Fiebig A, Nolan M, Lapidus A, Tice H, et al. Genome sequence of the mud-dwelling archaeon Methanoplanus limicola type strain (DSM 2279T), reclassification of Methanoplanus petrolearius as Methanolacinia petrolearia and emended descriptions of the genera Methanoplanus and Methanolacinia. Stand Genomic Sci. 2014;9: 1076. doi:10.4056/sigs.5138968
