# Supplementary material for: Wide range of metabolic adaptations to the acquisition of the Calvin cycle revealed by comparison of microbial genomes
Source: PLoS Comput Biol. 2021 Feb 8;17(2):e1008742. doi: 10.1371/journal.pcbi.1008742 (PMC7895386; doi:10.1371/journal.pcbi.1008742)
Supplement: S2 Table — The table lists all Pfams that match the search terms "photosynthesis", "photosynthetic", or "photosystem". The columns contain Pfam feature ID (’Feature’), feature name (’Name’), and feature description (’Description’; the DESC line from the Pfam HMM database). Given the occurrence of three of these Pfams in the photosynthetic organism Bradyrhizobium sp. ORS 278, photosynthesis capability was assigned to organisms with at least three of these Pfams. (PDF) [file pcbi.1008742.s003.pdf]

**S2 Table. Pfams associated with photosynthesis.**

| Feature | Name | Description |
|---|---|---|
| PF10643.9 | Cytochrome-c551 | Photosystem P840 reaction-centre cytochrome c-551 |
| PF02276.18 | CytoC_RC | Photosynthetic reaction centre cytochrome C subunit |
| PF11947.8 | DUF3464 | Photosynthesis affected mutant 68 |
| PF01716.18 | MSP | Manganese-stabilising protein / photosystem II polypeptide |
| PF00124.19 | Photo_RC | Photosynthetic reaction centre protein |
| PF03967.13 | PRCH | Photosynthetic reaction centre, H-chain N-terminal region |
| PF00223.19 | PsaA_PsaB | Photosystem I psaA/psaB protein |
| PF02605.15 | PsaL | Photosystem I reaction centre subunit XI |
| PF07465.13 | PsaM | Photosystem I protein M (PsaM) |
| PF05479.11 | PsaN | Photosystem I reaction centre subunit N (PSAN or PSI-N) |
| PF00737.20 | PsbH | Photosystem II 10 kDa phosphoprotein |
| PF02532.14 | PsbI | Photosystem II reaction centre I protein (PSII 4.8 kDa protein) |
| PF02533.15 | PsbK | Photosystem II 4 kDa reaction centre component |
| PF05151.12 | PsbM | Photosystem II reaction centre M protein (PsbM) |
| PF02468.15 | PsbN | Photosystem II reaction centre N protein (psbN) |
| PF04725.12 | PsbR | Photosystem II 10 kDa polypeptide PsbR |
| PF01405.17 | PsbT | Photosystem II reaction centre T protein |
| PF06514.11 | PsbU | Photosystem II 12 kDa extrinsic protein (PsbU) |
| PF07123.12 | PsbW | Photosystem II reaction centre W protein (PsbW) |
| PF06596.11 | PsbX | Photosystem II reaction centre X protein (PsbX) |
| PF06298.11 | PsbY | Photosystem II protein Y (PsbY) |
| PF00421.19 | PSII | Photosystem II protein |
| PF14870.6 | PSII_BNR | Photosynthesis system II assembly factor YCF48 |
| PF13326.6 | PSII_Pbs27 | Photosystem II Pbs27 |
| PF18240.1 | PSII_Pbs31 | Photosystem II Psb31 protein |
| PF05969.11 | PSII_Ycf12 | Photosystem II complex subunit Ycf12 |
| PF00796.18 | PSI_8 | Photosystem I reaction centre subunit VIII |
| PF02427.17 | PSI_PsaE | Photosystem I reaction centre subunit IV / PsaE |
| PF02507.15 | PSI_PsaF | Photosystem I reaction centre subunit III |
| PF03244.14 | PSI_PsaH | Photosystem I reaction centre subunit VI |
| PF01701.18 | PSI_PsaJ | Photosystem I reaction centre subunit IX / PsaJ |
| PF01241.18 | PSI_PSAK | Photosystem I psaG / psaK |
| PF10657.9 | RC-P840_PscD | Photosystem P840 reaction centre protein PscD |

The table lists all Pfams that match the search terms "photosynthesis", "photosynthetic", or "photosystem". The columns contain Pfam feature ID ('Feature'), feature name ('Name'), and feature description ('Description'; the DESC line from the Pfam HMM database). Given the occurrence of three of these Pfams in the photosynthetic organism *Bradyrhizobium* sp. ORS 278, photosynthesis capability was assigned to organisms with at least three of these Pfams.
