# Supplementary material for: Wide range of metabolic adaptations to the acquisition of the Calvin cycle revealed by comparison of microbial genomes
Source: PLoS Comput Biol. 2021 Feb 8;17(2):e1008742. doi: 10.1371/journal.pcbi.1008742 (PMC7895386; doi:10.1371/journal.pcbi.1008742)
Supplement: Attachment — Submitted filename: response_to_reviewers_revision_1_20201217_1205.pdf [file pcbi.1008742.s010.pdf]

Reviewer #1

none

Reply: We are grateful for your interest in our work.

Reviewer #2

Authors are presenting an extensive bioinformatical analysis of Calvin-Benson cycle and the metabolic adaptation of closely related pathways. The presented study provides valuable information both for other theoretical approaches as well for molecular engineering and biotechnological applications. However, there are several issues which should be addressed:

Reply: We are grateful for your recognition of the value this study provides and for taking your time to identify several outstanding issues.

Major comments:

1) Cyanobacteria diverge strongly from other bacteria due to an ancient evolutionary emergence and were therefore excluded from further analysis.

Differences in adaptation towards Calvin cycle among cyanobacteria themselves, as well as to other photosynthetic bacteria, might be the most interesting results also from the biotechnological point of view. The question is if their analysis would have to be done separately and what would be the control for each species (closest bacterium/cyanobacterium, the simplest/oldest cyanobacterium, …)

Reply: Yes, it is our conclusion that the analysis of Cyanobacteria would have to be done separately. As hinted in the first version of the manuscript, the ancient origin of Cyanobacteria make them very different from their "closest relatives", which belong to completely different bacterial groups, thus making it difficult to pinpoint adaptations that are specific for the Calvin cycle and not specific to the phylum of Cyanobacteria, such as photosynthetic reaction centers, chlorophyll and phycobilin synthesis, aquatic lifestyle (for many genomes), etc. We saw these things in an earlier version of the analysis (commit 9ad7914 and back) and had to make a decision whether to exclude Cyanobacteria or try to tweak the analysis to facilitate their inclusion. We opted for exclusion as it was the cleanest solution. Unfortunately, we did not explain our reasons behind excluding Cyanobacteria well enough in the manuscript. Thank you for highlighting this issue! Therefore we have now added a thorough explanation before revealing the exclusion of Cyanobacteria (lines 143-158). The explanation includes quantitative data to support our claim that Cyanobacteria are much further evolved compared to their closest relatives than are other microbes in our dataset (median distance 0.74 compared to 0.14, $p \approx 1.5 \cdot 10^{-83}$, Wilcoxon rank sum test). Put simply, the purpose of our manuscript is to investigate early adaptations to the Calvin cycle, and Cyanobacteria do not fit that purpose. In order to make use of potential adaptations to the Calvin cycle unique to Cyanobacteria, a different approach than the one described here would be necessary, for example by filtering out genes that are not more than phylogenetic signatures of Cyanobacteria as an organism group, or by doing a direct comparison between Cyanobacteria and other bacteria that have the Calvin cycle. Another option is to follow horizontal gene transfer processes such as the one that may have provided *Prochlorococcus* and *Synechococcus* with proteobacterial Rubisco (Delwiche and Palmer, 1996; doi:10.1093/oxfordjournals.molbev.a025647). Again, however, that would not fit our theme of comparing bacteria with and without the Calvin cycle. Finally, we added a reference to the 2012 study by Beck *et al.* that already made a thorough investigation of cyanobacterial metabolism using multiple genomes (doi:10.1186/1471-2164-13-56).

2) „Photorespiration salvage did not appear to be adapted specifically for the Calvin cycle.“

Excluded cyanobacteria might have shown a very different picture here. It is known that isozymes have a significant impact on metabolic adaptation, namely the second reaction of photorespiration is producing a toxic compound which is dealt with by 2-4 isozymes of phosphoglycolate phosphatase in cyanobacteria. And that is not the only isozyme within photorespiratory pathway. Authors mentioned isozymes for Calvin-Benson cycle and glycolysis but not for photorespiration; I guess that there are none in other bacteria? This whole study is about contrast and comparison, I cannot imagine a bigger contrast or maybe a surprise if cyanobacteria would have been included in this analysis.

Reply: Thank your for pointing this out. We looked at how many CBB-positive genomes have more than one copy of phosphoglycolate phosphatase and found 157 such genomes, almost exclusively in Gammaproteobacteria and Alphaproteobacteria. This means that most CBB-positive bacteria in our dataset do not have multiple copies of phosphoglycolate phosphatase (some even have none), but a select few could be using isozymes just like in Cyanobacteria. We expanded the paragraph about photorespiration using these new data (lines 431-444), and also added a reference to the 2014 study by Jablonsky *et al.* that identified the benefits of phosphoglycolate phosphatase isozymes in *Synechococcus elongatus* PCC 7942 (doi:10.1371/journal.pone.0105292).

3) Authors picked the presence of large subunit of RuBisCO and Prk as an evidence of Calvin cycle. However, is it just their assumption or undisputed evidence for the existence of fully functional Calvin cycle?

Reply: We do make the assumption that the presence of the large subunit of Rubisco and Prk will enable Rubisco carbon fixation by connecting to other metabolic pathways existing in the organisms. We do not test for those connections explicitly. Given the chances of missing such connections due to incomplete genomes and other potential errors discussed in the text, we think it is best to only look for Rubisco and Prk, and then assume that there are other adaptations present to allow their utilization. It is clear that this is the case, as seen for the enrichment of other Calvin cycle genes in CBB-positive genomes. In any case, thank you for pointing out that this assumption was not clearly mentioned. We see that it could be misunderstood that we may have ensured an integration with other metabolic pathways, which we did not. To address this problem, we now clearly state that *"We assumed that the presence of both Rubisco and Prk in 1,490 of the genomes (6.0%) indicated a complete Calvin cycle and thus CBB-positive classification."* on lines 123-124. Furthermore, we also mention that we did not include the small subunit in the requirements (lines 119-121), because the small subunit does not have catalytic activity and is only present in form I Rubisco, as reported for example in the newly added reference to Tourova and Spiridonova 2009 (doi:10.1134/S0026893309050033). Additionally, we acknowledged already in the first version of the manuscript that Rubisco and Prk presence in Archaea may be the reductive hexulose-phosphate pathway (lines 162-163 in the new version).

4) Presented algorithm for Calvin cycle identification has an accuracy around 75% and is compared basically to flipping a coin (50/50). It would be more valuable to compare its accuracy to some other algorithm(s) from the same field.

Reply: Thank you for the suggestion. We agree that a comparison to other algorithms would be more informative and have therefore added three references where similar datasets and algorithms were used (lines 268-273). Those algorithms achieved 86-94% accuracy, thereby establishing that our algorithm has some room for improvement. This puts the following discussion about what could be limiting the accuracy into a relevant context and we think this is a valuable addition to the manuscript.

5) Analysis presented on Fig. 5 is missing one of key exit point out of Calvin cycle, the phosphoketolase pathway. It would be interesting to see the importance of this pathway in comparison to lower EMP glycolysis as it prevents the decarboxylation.

Reply: Phosphoketolase (Xfpk) was previously excluded in Fig 5 because the enzyme was filtered out in the random forest analysis. On the initiative of reviewer #3 we have now switched to displaying consensus ranks in Fig 5 instead of random forest importance values. Therefore it was possible to add Xfpk. However, Xfpk ranked poorly and appeared not to be a Calvin cycle-specific adaptation in the context of our analysis. Note that we rerouted the Calvin cycle in Fig 5 in conjunction with the addition of Xfpk, and therefore also changed the maps in Figs 1 and 6. We have added a paragraph about Xfpk at the end of the *"Core Calvin cycle enzyme genes…"* section (lines 385-390). While Xfpk ranks poorly, it is also quite rare (average copy number ~0.07 to 0.2).

6) Rather technical language, paragraphs not linked together, and usage of uncommon synonyms made the reading difficult to follow what authors wanted to say.

Reply: Thank you for making us aware of these issues. We want our study to be accessible by a wide audience, so we have made attempts to improve the manuscript based on your comments. First, we feel that much of the technical language at the beginning of the Results and discussion section is necessary, *i.e.* for discussing the limitations of the methods. There we have to use some rather technical terms to be precise. However, we have made some attempts to improve the readability throughout the manuscript. First, prompted by reviewer #3 we have removed all references to ranks within the individual methods and instead use the consensus rank whenever rank is mentioned. Second, after first introducing the technical term "genetic features" clearly on line 177, we switch to "genes" from lines 180-181 and onwards (excluding Materials and methods and Supporting information), since "genes" should be more straightforward for biologists in general. Next we added a few short sentences within Results and discussion that link the sections. Such sentences were previously lost in attempts to reduce the word count. We hope that the addition of those sentences will make the reading experience more coherent. The usage of uncommon synonyms is probably also a symptom of overzealous word count reduction. These words have now been replaced with more common synonyms or phrases. We feel that this change has made the message of at least a few sentences more clear. Finally, we have made extensive edits to most parts of the text to make it less technical and easier to read.

Minor comments:

1) „Photorespiration salvage“ – this phrase is not commonly used, probably due to the fact that photorespiratory pathway has other roles than „just“ salvaging phosphoglycolate. If authors want to use it, I would recommend to use quotation marks for the word salvage.

Reply: Thank you for spotting this and making us aware of the uncommon phrase. We have removed “salvage” and now only write “photorespiration”. However, we do write about 2-phosphoglycolate “salvage” once (line 456), but added the quotation marks as suggested.

2) Few little grammar errors here and there but nothing uncommon for the manuscript of this length.

Reply: We have gone through the manuscript thoroughly and corrected any errors we could identify. Additionally, differences in abbreviations and minor alignment errors have been amended in the figures.

Reviewer #3

Although improving the efficiency of the Calvin cycle in plants has been a long-term goal for genetic engineers, only modest progress has been made so far. In this work, Asplung-Samuelsson and Hudson try to take a step back and explore the phylogenetic tree for genetic adaptations that have been relevant for this pathway using strong statistical methods. The results of this analysis could prove very valuable for metabolic engineers working on autotrophic growth and therefore might help the global effort to mitigate climate change.
The workflow is well described in the text, and the analysis code is freely available on GitHub, which is commendable.

Reply: We do hope that this analysis may serve as a genomic reference work and provide novel views of Calvin cycle metabolism. Thank you for taking the time to read the manuscript and provide valuable feedback.

Major comment:

It's not very clear why three different methods were used for ranking the features. Presumably, using the consensus ranking of all 3 prevents certain outliers that could come from one of the three methods and increases the confidence of the ranking. Whatever the reason, showing results from different classifiers throughout the text is more confusing than helpful. It would be easier to follow of a single ranking system was used (and justified) throughout the results section. For example, in figure 5, colors indicate RF importance while thickness indicates enrichment score. What about the ACE scores? Why not show the consensus ranking instead (e.g. color coded only)?
In the text itself, ranking from different systems is often mentioned side by side, which is not very helpful. The fact that ACE provides many correlations depending on the sub-tree only adds to the confusion. Also, having so many scores gives the impression that some results are cherry-picked.

Reply: This is a very good point. Finding a good way to present the results from the three different methods has been a challenge for us. Thank you for providing ideas on how to improve the presentation. We have now added a justification for using the three methods on lines 196-211, which includes the purpose of removing outliers, as you assumed, but also mentions the intention to probe every gene and aspect of adaptation thoroughly by using different approaches, as well as the benefit of having different metrics that may explain why a particular gene has a good rank. Furthermore, we agree that individual method ranks are confusing and have therefore removed them. Now only the consensus rank is used, but we do keep references to gene copy numbers from the enrichment analysis and correlations in specific subtrees, because we think it is necessary to know why genes rank they way they do, and also to be reminded that the data are complex and could vary between organism groups. Additionally, we acknowledge that we cannot explore every potential adaptation to the Calvin cycle and explain our focus before the first paragraph *"Core Calvin cycle enzyme genes…"* by adding the paragraph *"Below we report on what we identified as the most prominent, interesting, and relevant biological patterns in the dataset. We consider adaptations in the top 10%, i.e. the top 1250 genes, to have good consensus ranks, while others are poor, but the focus is mainly on the top 200 genes. There are other adaptations with a narrower scope that are not discussed here, but can be found in the supporting information."* on lines 278-

282. We have also followed your advice to show consensus ranks in Fig 5 instead of random forest importance values. We have kept line thickness because we think this is useful information to get a quick idea about whether an enzyme is important because there are more copies of it or fewer (this is referenced in the discussion of the core Calvin cycle enzymes, ED/OPP pathways, as well as photorespiration). Fig 5 saw some rather extensive rework to make it more relevant by adding certain reactions that were no longer filtered out, adding Xfpk (see major comment 5 from reviewer #2), and removing reactions, *e.g.* those in the ED pathway that did not have phosphorylated metabolites. We think these changes have improved the manuscript by making it less confusing and more coherent.

Minor comments:

• The definition of "consensus rank" is not given before it is first used in line 171.

Reply: Thank you for spotting this oversight. We have now made sure that the consensus rank is described at first mention (lines 206-208).

• Line 167-169: The Spearman correlation between ACE and random forest is higher than the other two pairs, but the p-value is not lower. I assume it is because there are only 1200 features for the RFs. Perhaps indicating "n" as well as p-value and "r" would help readers wondering about the apparent inconsistency.

Reply: We have added the number of data points (n) as suggested (lines 192-193). For ACE vs RF it is n = 1,192, enrichment vs RF is n = 1,194, and ACE vs enrichment is n = 11,731. The very low p-value achieved by ACE vs enrichment is clearly related to the large number of data points, and the p-value and correlation coefficients seem to change as expected for the two comparisons that have roughly the same number of data points.

• Figure 4: it's not clear to me why the fact that most archea are CBB- explains the negative correlation with CbbQ.

Reply: Most Archaea with CbbQ are CBB-negative, which explains why there is a negative correlation between CbbQ and CBB-positive status. We have tried to change the wording to make the sentence in the Fig 4 caption more clear on lines 264-265. We also added further explanations that brown indicates CBB-negative genomes and cyan indicates CBB-positive genomes in Fig 4 on lines 257-258 and line 265 of the Fig 4 caption.

• Line 250-251: I suggest changed "compared to glycolysis" with something more specific such as "compared to heterotrophic growth on glycolytic carbon sources such as glucose" (FBP and TKT are not even part of glycolysis).

Reply: Thank you for the suggestion. We have replaced "compared to glycolysis" with "compared to heterotrophic growth on glycolytic carbon sources such as glucose" on lines 295-296.

• Figure 5: since the colorbar is scaled to be 0-1 after the log10 transform, the exponent of the log-transform is irrelevant (it is enough to say that it is logarithmic scale).

Reply: As suggested, we have switched to denoting it a logarithmic scale (line 303). Furthermore, we decided to improve the color bar in Fig 5 by adding tick marks on a logarithmic scale to show the actual rank of each color. We also plotted points above the color bar to indicate what consensus ranks are represented by enzymes, which gives an idea of the distribution of ranks and how many of each color should be present in the map.

• Line 324-330: the Entner-Doudoroff pathway does not convert glucose-6-phosphate to ribulose-5-phosphate (Gnd is not part of that pathway). And although Zwf can be considered part of the pathway, it is not exclusive to the ED. I suggest writing about "the ED and OPP pathways" together since they are difficult to distinguish in the context of this analysis.

Reply: Thank you for identifying this misunderstanding. We have corrected mentions of "ED" with "ED and OPP" throughout lines 367-371.
